# Inferring interactions in complex microbial communities from nucleotide sequence data and environmental parameters

**DOI:** 10.1371/journal.pone.0173765

**Published:** 2017-03-13

**Authors:** Yu Shang, Johannes Sikorski, Michael Bonkowski, Anna-Maria Fiore-Donno, Ellen Kandeler, Sven Marhan, Runa S. Boeddinghaus, Emily F. Solly, Marion Schrumpf, Ingo Schöning, Tesfaye Wubet, Francois Buscot, Jörg Overmann

**Affiliations:** 1 Leibniz Institute DSMZ-German Collection of Microorganisms and Cell Cultures, Inhoffenstraße 7B, D-38124, Braunschweig, Deutschland; 2 Department of Terrestrial Ecology, Institute of Zoology, University of Cologne, Zülpicher Straße 47b, D-50674 Köln, Deutschland; 3 Institute of Soil Science and Land Evaluation, Soil Biology Section (310b), University of Hohenheim, Emil-Wolff-Straße. 27, D-70593 Stuttgart, Deutschland; 4 Max-Planck-Institut für Biogeochemie, Hans-Knöll-Straße 10, D-07745 Jena, Deutschland; 5 Department of Soil Ecology, Helmholtz Centre for Environmental Research - UFZ, Theodor-Lieser-Straße 4, D-06120, Halle/Saale, Deutschland; 6 German Center for Integrative Biodiversity Research (iDiv) Jena Halle Leipzig, Deutscher Platz 5e, 04103 Leipzig, Deutschland; Wageningen University, NETHERLANDS

## Abstract

Interactions occur between two or more organisms affecting each other. Interactions are decisive for the ecology of the organisms. Without direct experimental evidence the analysis of interactions is difficult. Correlation analyses that are based on co-occurrences are often used to approximate interaction. Here, we present a new mathematical model to estimate the interaction strengths between taxa, based on changes in their relative abundances across environmental gradients.

## Introduction

The composition of microbial communities is a key driver of ecological processes [1–3]. Changes in the abundances of species can occur in response to abiotic selection pressures, neutral assembly processes and be affected by organismal interactions. Biotic interactions are multifarious (competition, mutualism, commensalism, amensalism, and antagonism including parasitism and predation) and may have positive, negative, or neutral consequences for either one or both interacting partners. In most of the cases, outcomes of interactions between two species may be asymmetric in terms of abundance, e.g. predators will negatively affect the abundance of prey, but prey consumption will increase the abundance of the predator. As another example, a strong competitor will decrease the abundance of a neighboring species, but the latter may have no net effect on the former. Moreover, the strength of the influence can also differ, e.g. a preferred prey receives stronger top-down control than a general prey. Conversely, the prey could exert only a weak positive influence on the predator if it is of minor food quality.

While biodiversity studies of higher organisms have yielded large sets of multitrophic data and enabled a comprehensive analysis of interactions [4], the peculiarities of microbial communities render the detection and study of interactions very challenging. Whereas potential pairwise interactions among microorganisms can be studied directly in the laboratory, the determination of interactions in large and complex biotic communities under natural conditions is limited. Soil ecosystems in particular harbor extremely diverse microbial communities. The diversity of bacteria may reach 10^4^ species [5] and average cell numbers of 10^10^ per gram of soil [6]. This results in highly complex networks of coexisting microbes [7]. Secondly, only a minority (0.1%–0.001%) of the microbial diversity has been cultivated to date [8], which precludes experimental analysis of the majority of interactions in the laboratory. Thirdly, the heterogeneous structure of the soil habitat at the microscale represents an additional methodological and conceptual challenge. Due to the complexity of soil structure, microbial cells typically occur in a non-random fashion in clusters with cell-to-cell distances of only 1-10 *μ*m, which is the distance at which interactions between microbes is assumed to take place by either direct cell to cell contact or by diffusion limit of chemical substances. However, at this spatial scale, it is impossible to study organismal interactions simply by passive observations as in macroorganisms. In contrast, most studies of microbes in complex habitats typically are conducted through destructive sampling methodology, precluding the consecutive analysis of the same microbial community over time, which would be a prerequisite to applying the established discrete-time Lotka-Volterra models [9]. As a result, the lack of knowledge on species interactions has remained one of the major shortfalls in understanding the drivers of ecosystem functions [10]

A major step forward in analyzing the composition of microbial communities in the environment has been the advent of high-throughput next-generation sequencing technology for microbial DNA or RNA extracted from the environment. This enables the determination of the relative abundance of many taxa per sample and the analysis of co-occurrence and correlation patterns, which have been suggested as proxies for species interactions [7, 11]. However, the latter approach may only reflect similar responses of different species towards environmental pressures rather than direct interaction and cannot resolve interactions that are asymmetric. Also, several important properties of interactions can not be captured by correlations. Firstly, interactions may be asymmetric such that taxon A may influence B negatively but B influences A positively. In contrast, the correlation of A with B is the same as B with A, hence symmetric. Secondly, the direction of interaction may be different, e.g., A influences B positively, but under some circumstances, A may influence B negatively. In contrast, the concept of correlation analysis requires that the abundance relationship of two taxa remains consistent throughout: if A increases, B always either increases too (positive correlation) or decreases (negative correlation).

Fisher et al. [9] established the LIMITS algorithm to infer the interaction among microbial species from the time series data based on the discrete time Lotka-Volterra Model. Bucci et al. [12] also suggested utilizing the generalized Lotka-Volterra equation with time-dependent perturbation to analyse the interaction from the time series data. However, both of these approaches can not be used for series of cross-sectional data which originate from samples along gradients in environmental parameters but do not have a temporal dimension. Therefore, here, we present a novel method which is based on generalized Lotka-Volterra models and allows to quantify interactions from high-throughput microbial sequence data derived from cross-sectional samples for which also larger datasets on environmental parameters are available. This method does not rely on data obtained during different time points, since the strength and direction of interactions between partners are also a function of gradients in abiotic environmental parameters, for example of temperature [13], nutrient conditions [14] or other factors such as soil moisture, mineral content, pH or osmolarity [15–17]. Biswas et al. [18] developed a Poisson-multivariate normal hierarchical model to analyse interactions, taking into account also the environmental gradients. In this work, the environmental parameters are included into the parameters of the Poisson function which is used to express the species abundance distribution. But, this calculation still represents a type of correlation analysis. Hence, the interaction matrix is symmetric and can not account for asymmetric interactions. On the contrary, our method is fundamentally different from correlation analysis and can analyse asymmetric interactions. Sugihara et al. [19] developed a causality test (CCM) to infer the causal link between the time-series variables which are not correlated. CCM analysed locally the historical time trajectory of the different variables and measured the extent to which the historical record of one variable can reliably estimate states of another variable. CCM can infer whether these variables are strongly linked to each other without consideration of environmental gradients. However, CCM can not account for the asymmetry property in the interaction relationship. In contrast, our approach can address this issue in the sense of changing environmental gradients but does not take into account the time series data. The basic assumptions of our model are that (i) interactions lead to changes of abundances within individual species, and that (ii) the abundances of a species are a function of the species abundances of the remaining community as well as of environmental parameters. We first describe the theoretical foundation of our framework and then suggest a numerical implementation calculating the direction and strength of interactions between two pairs of taxa, which depend on the quality criteria assigned to the determination of environmental parameter values and relative taxon abundances retrieved from high-throughput sequences. We further present methods to calculate single representative interaction values and to determine their robustness by appropriate statistical tools such as random sampling. Finally, we discuss the potentials of our approach.

## Methods

The aim of our approach is to determine the interaction coefficient *β*_*ij*_, which quantifies the interaction strength of species *j* on species *i*. We firstly present the theoretical basis for our approach. Secondly, depending on the precision quality of the input data, we suggest numerical calculation approaches estimating $\beta_{ij\alpha }^{k}$ for a given environmental parameter *α* and a given sample *k*. The result is a two-dimensional matrix of numerical $\beta_{ij\alpha }^{k}$ values with *m* (*m* is the number of environmental parameters) rows and *N* (*N* is the number of samples) columns for each pair of species *j* having interaction influence on species *i*. Thirdly, we addressed the issue about data structure and the precision of the calculation. We fourthly present several strategies to summarize the $\beta_{ij\alpha }^{k}$ values into a global *β*_*ij*_ value. Finally, we present an estimate for the robustness of *β*_*ij*_ based on random sampling and the addition of numerical noise to the input data.

### Theoretical deductions

Species abundances change as a response to changing environmental conditions and abundances of interacting organisms. Therefore the information on the direction and strength of biotic interactions must be stored in the change of species abundances and hence can be extracted from that.

The basic idea of this methods is to analyze the influence from other interacting species abundance on the rate of change of specific species abundance in the sense of environmental gradients.

**Fig 1:**
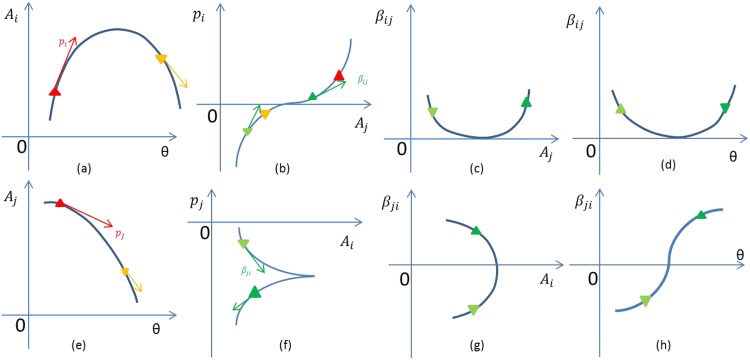
The sketch on the analysis workflow.

The Fig 1 shows the detailed conceptual idea of this approach and the sketch on the analysis workflow of obtaining *β*_*ij*_ interaction values from values of relative abundances of taxa and environmental parameters obtained from a set of sampling sites. The change of species abundance *A*_*i*_ and *A*_*j*_ with respect to one environmental parameter *θ* are presented as a curve in subplots (a) and (e), respectively. In subplot(a), the rate of change *p*_*i*_ of *A*_*i*_ with respect to Θ was determined by calculating the slope of the tangent vector on each curve point. It reflects the influence of environmental parameter Θ on the change of abundance *A*_*i*_ across the environmental gradients. On the red triangular point, the slope is positive, means the rate change at this point (corresponding to one *θ* value) is positive, the abundance *A*_*i*_ has the increasing tendency at that gradient value of Θ. Similarly, the orange triangular point has a negative rate of change reflecting the decreasing tendency of *A*_*i*_ under these (larger) values of the gradient of Θ. Based on the calculated *p*_*i*_ for each values of *θ* and the abundance curve of *A*_*j*_, the relationship between *p*_*i*_ and *A*_*j*_ is shown in subplot (b) (the red triangular has higher abundance values of *A*_*j*_ than the orange triangular). Then, the influence of *A*_*j*_ on the change of *p*_*i*_ can be denoted by the tangent vector (light green triangular and dark green triangular are the two example points). The slope of this new tangent vector stand for the rate of change of *p*_*i*_ with respect to *A*_*j*_, and it is denoted as *β*_*ij*_. *β*_*ij*_ is the interaction influence from species *j* on species *i* in the sense of *θ*. The relationship of *β*_*ij*_ and *A*_*j*_ is presented in subplot (c). Due to the relationship of *A*_*j*_ and *θ* in subplot(b), the relationship between *β*_*ij*_ and *θ* is shown in subplot (d). The subplot (d) inform us the change of interaction influence *β*_*ij*_ in different environmental conditions which are corresponding to the different value of *θ*. Similarly, we can do the same rate change analysis for *A*_*j*_ the corresponding results are shown in subplots (f), (g) and (h).

By analyzing the rate of change of species abundance, *p*_*i*_, and the change of *p*_*i*_ with respect to other species, we can deduce the interaction influence between them. From this figure, *β*_*ij*_ is positive, suggesting that the species *j* has a positive interaction influence on species *i*. Conversely, *β*_*ji*_ is negative, suggesting that the species *i* has a negative interaction influence on species *j*. Although one pair of species shares the same interaction relationship, the effect of the interaction on both of them could be different, not only in the direction but also on the strength. Moreover, based on the subplot (a) and (e), there is no clear correlation between *A*_*i*_ and *A*_*j*_. This indicates that the correlation is not equal to interaction, hence, the analysis of correlation between the species abundances is not suitable to infer the interaction relationship between them. The parameter *β*_*ij*_ is chosen in analogy to the Lotka-Volterra equation. The details are explained as follows.

Assume that the abundance *A*_*i*_ of species *i* is the smooth function of interacting species *A*_*j*_(*j* ≠ *i*) and the environmental parameters Θ_*α*_, *α* = 1, 2, ⋯, *m* (in case of soil, for example, soil moisture, pH, nutrient contents; where changes in time are available, even time *t* can be used). In a two species interaction system, the change in abundance of both species in response to the change of environmental parameters and biotic interactions are

$$\begin{array}{ccc} \frac{dA_{i}}{d\Theta } & = & S_{i}\left(A_{i}\right)+I_{ij}\left(A_{i}A_{j}\right)\\ \frac{dA_{j}}{d\Theta } & = & S_{j}\left(A_{j}\right)+I_{ji}\left(A_{j}A_{i}\right) \end{array}$$ (1)

where *S*_*i*_ can be treated as the solitary part of species *i*, i.e. change of *A*_*i*_ independent of any influence from other species, it is also influenced by the environmental parameter. The derivative $\frac{dA_{i}}{d\Theta }$ is the rate of change of *A*_*i*_ with respect to the change of values of Θ. *I*_*ij*_ is the influence from species *j* on species *i*, which is a function of *A*_*i*_, *A*_*j*_, and also Θ. In different environmental conditions, *I*_*ij*_ will be different. Accordingly, the interaction can be analysed based on the gradient of Θ and will demonstrate how the interaction levels change across the different environmental conditions. Note that this can be asymmetric, i.e., *I*_*ij*_ ≠ *I*_*ji*_. Thus, the effects of the interaction between species *i* and *j* could be different with respect to the rate of change of *A*_*i*_ and of *A*_*j*_. Note also that the exact mathematical form of *S*_*i*_, *S*_*j*_ is often unknown due to lack of suitable experimental data, but can be approximated to follow the Monod equation or logistic equation [20–22]. To analyze the interaction, *I*_*ij*_ and *I*_*ji*_ need to be resolved. In order to calculate the change of $\frac{dA_{i}}{d\Theta }$ with respect to *A*_*j*_ we remove the unknown part *S*_*i*_, as follows

$$\begin{array}{ccc} \frac{d(\frac{A_{i}}{d\Theta })}{dA_{j}} & = & \frac{dI_{ij}\left(A_{i},A_{j}\right)}{dA_{j}} \end{array}$$ (2)

The interaction information is contained on the right-hand side of the equation. For calculating the interaction numerically, we need the concrete mathematical expression of *I*_*ij*_. For simplification, we assume:

$$\begin{array}{c} I_{ij}=\beta_{ij}A_{i}A_{j} \end{array}$$ (3)

Because *A*_*i*_ is a multivariate function of Θ_*α*_, the rate of change of *A*_*i*_ with respect to Θ_*α*_ which is also a multivariate function of Θ_*α*_ can be expressed by using the partial derivative:

$$\begin{array}{c} p_{i\alpha }=\frac{\partial A_{i}}{\partial \Theta_{\alpha }} \end{array}$$ (4)

As the change of *p*_*iα*_ is affected by *β*_*ij*_, the information of *β*_*ij*_ is stored in the change of *p*_*iα*_.

With the approximation of Eq (3), *β*_*ij*_ can then be estimated as:

$$\begin{array}{c} \beta_{ij\alpha }=\frac{\partial p_{i\alpha }}{\partial A_{j}}\frac{1}{A_{i}} \end{array}$$ (5)

We define the interaction level *β*_*ij*_ as the rate of change of *p*_*iα*_ with respect to the abundance *A*_*j*_ of species *j*. Thus, the interaction level *β*_*ij*_ will be the smooth functions of species abundances *A*_*i*_, *A*_*j*_ and the environmental gradients stored in Θ_*α*_.

The above concept of using changes in species abundance for the calculation of interaction values is analogous to time-dependent generalized Lotka-Volterra equations (predator-prey equations):

$$\begin{array}{ccc} \frac{dA_{1}}{dt} & = & r_{1}A_{1}\left(1-\frac{A_{1}}{K_{1}}+\beta_{12}\frac{A_{2}}{K_{1}}\right)\\ \frac{dA_{2}}{dt} & = & r_{2}A_{2}\left(1-\frac{A_{2}}{K_{2}}+\beta_{21}\frac{A_{1}}{K_{2}}\right) \end{array}$$ (6)

Here, the parameters *r*_1_, *r*_2_ are the growth rate, *K*_1_, *K*_2_ are the carrying capacity of the system [22]. Comparison of Eq (6) to Eq (1) demonstrates that: Θ is equivalent to the time parameter *t*, and $I_{12}=\frac{r_{1}}{K_{1}}\beta_{12}A_{1}A_{2}$. Incorporation of $\frac{r_{1}}{K_{1}}$ into *β*_12_ yields the:

$$\begin{array}{c} I_{12}=\beta_{12}^{*}A_{1}A_{2} \end{array}$$ (7)

By using Eq (5), we can estimate $\beta_{12}^{*}=\frac{d(\frac{A_{1}}{dt})}{dA_{2}}\frac{1}{A_{1}}$, which represents the estimation of the interaction level from the species abundance change in the Lotka-Valterra equation.

### Numerical determination of $\beta_{ij\alpha }^{k}$ values

In microbial ecology, absolute abundances of individual cells can usually not be determined for all taxa at all taxonomic hierarchy levels. With high-throughput sequence data, the abundance of a given taxon in sample *k* is actually given as a relative abundance value, which is the number of sequences reads assigned to that taxon among all sequence reads in the respective sample *k*. The determination of the relative abundance value of a specific taxon by high-throughput sequencing is not error-free. Small but uncontrollable variations in nucleic acid extractions, cDNA synthesis (in case RNA is extracted), amplicon primer ligation, and sequencing runs on high-throughput sequencers add uncertainty to the estimated relative abundance value. In the case of abundant taxa, typically at class or phylum level, the uncertainty may encompass just a 1% to 10% error level [23]. However, for less abundant taxa at the level of genera or species (defined by 97% similarity of the 16S rRNA gene [24]), the error could be much larger (two–fold, own unpublished data).

Similarly, the determination of physicochemical environmental parameters from soil such as pH, soil moisture, carbon and nitrogen content, is accompanied by uncertainty errors mostly due to soil heterogeneity which may also be in the range of 1% to 15% (own unpublished data).

We refer to data with assumed low (1-10%) experimental error in the estimation of numerical input data, and describe how to numerically calculate $\frac{\partial A_{i}}{\partial \Theta_{\alpha }}$ and $\frac{\partial p_{i\alpha }}{\partial A_{j}}$ from a data set derived from different samples using the Taylor expansion [25].

If the samples are denoted by using the index *k* = 1, 2, ⋯ *N*, we denote $A_{i}^{k}$, $\Theta_{\alpha }^{k}$ as the abundance of species *A*_*i*_ and environmental parameter Θ_*α*_ in sample *k*, respectively. The rate of change of *A*_*i*_ with respect to Θ_*α*_ in sample *k* is defined as the partial derivative:

$$\begin{array}{c} p_{i\alpha }^{k}=\frac{\partial A_{i}^{k}}{\partial \Theta_{\alpha }^{k}} \end{array}$$ (8)

and the interaction level *β*_*ij*_ as the rate of change of $p_{i\alpha }^{k}$ with respect to species *j* abundance $A_{j}^{k}$ according to

$$\begin{array}{c} \beta_{ij\alpha }^{k}=\frac{\partial p_{i\alpha }^{k}}{\partial A_{j}^{k}}\frac{1}{A_{i}^{k}} \end{array}$$ (9)

$\beta_{ij\alpha }^{k}$ represents the interaction value characterizing the interaction influence of species *j* on species *i* accompanying the change in environmental parameter Θ_*α*_ in sample *k*. This allows analyzing the interaction of species *j* on species *i* for different environmental parameters, and its change across different environmental conditions.

Note that for this part of the analysis, the numerical calculation of the partial derivative $\frac{\partial A_{i}^{k}}{\partial \Theta_{\alpha }^{k}}$ and $\frac{\partial p_{i\alpha }^{k}}{\partial A_{j}^{k}}$ normally requires to fix the values of the environmental parameters Θ_*α*_ to be the same in the other samples as in sample *k*. This mathematical requirement can not be fulfilled as real world samples differ typically at the same time in both species abundances and values of environmental parameters. We, therefore, make use of the Taylor expansion of multivariate functions to obtain the accurate numerical calculation when both environmental parameter values *α* and species abundances *A* change across samples *k* simultaneously. As addressed above, $A_{i}^{k}$ is a multivariate function of environmental parameters {Θ_*α*_} and other interacting species $A_{j}^{k}$.

Between two different samples, the abundance of species *i* can be expressed as $A_{i}^{k}\left(\Theta^{k}\right)$ and $A_{i}^{l}\left(\Theta^{l}\right)$. Here, Θ^*k*^ and Θ^*l*^ are the corresponding environmental parameters in sample *k* and sample *l*. Using the Taylor expansion, the difference between $A_{i}^{k}\left(\Theta^{k}\right)$ and $A_{i}^{l}\left(\Theta^{l}\right)$ can be expressed as

$$\begin{array}{ccc} A_{i}^{l} & & =\sum_{r_{1}=0}^{\infty }\cdots \sum_{r_{d}=0}^{\infty }\sum_{s_{1}=0}^{\infty }\cdots \sum_{s_{m}=0}^{\infty }\frac{{\left(A_{1}^{l}-A_{1}^{k}\right)}^{r_{1}}\cdots {\left(A_{d}^{l}-A_{d}^{k}\right)}^{r_{d}}{\left(\Theta_{1}^{l}-\Theta_{1}^{k}\right)}^{s_{1}}\cdots {\left(\Theta_{m}^{l}-\Theta_{m}^{k}\right)}^{s_{m}}}{r_{1}!\cdots r_{d}!s_{1}!\cdots s_{m}!}\\ & & \cdot \left(\frac{\partial^{r_{1}+\cdots +r_{d}+s_{1}+\cdots +s_{m}}A_{i}^{l}}{\partial A_{1}^{r_{1}}\cdots \partial A_{d}^{r_{d}}\partial \Theta_{1}^{s_{1}}\cdots \partial \Theta_{m}^{s_{m}}}\right)\left(A_{1}^{k},\cdots ,A_{d}^{k},\Theta_{1}^{k},\cdots ,\Theta_{m}^{k}\right) \end{array}$$ (10)

Using only the linear part of the approximation simplifies the formula as follows:

$$\begin{array}{ccc} A_{i}^{l}\left(\Theta^{l}\right)-A_{i}^{k}\left(\Theta^{k}\right) & = & \sum_{\alpha }\left(\Theta_{\alpha }^{l}-\Theta_{\alpha }^{k}\right)\frac{\partial A_{i}^{k}\left(\Theta \right)}{\partial \Theta_{\alpha }^{k}}+\sum_{j\neq i}\left(A_{j}^{l}-A_{j}^{k}\right)\frac{\partial A_{i}^{k}\left(\Theta \right)}{\partial A_{j}^{k}}\\ & = & \sum_{\alpha }\left(\Theta_{\alpha }^{l}-\Theta_{\alpha }^{k}\right)p_{i\alpha }^{k}+\sum_{j\neq i}\left(A_{j}^{l}-A_{j}^{k}\right)p_{ij}^{k} \end{array}$$ (11)

The first part addresses the influence from the change of environmental parameters whereas the second part addresses the influence from the change of other species abundances. We fix the sample *k*, and let the sample *l* run across all remaining samples, in order to then estimate $p_{i\alpha }^{k}$ from linear regression. Actually, the term $p_{ij}^{k}$, which addresses the rate of change of *A*_*i*_ with respect to *A*_*j*_ in sample *k*, can also be estimated as the by-product.

Because $p_{ij}^{k}$ is not necessary in the following $\beta_{ij\alpha }^{k}$ calculation, we reduce the model in Eq (11) to

$$\begin{array}{ccc} A_{i}^{l} & & =\sum_{s_{1}=0}^{\infty }\cdots \sum_{s_{m}=0}^{\infty }\frac{{\left(\Theta_{1}^{l}-\Theta_{1}^{k}\right)}^{s_{1}}\cdots {\left(\Theta_{m}^{l}-\Theta_{m}^{k}\right)}^{s_{m}}}{s_{1}!\cdots s_{m}!}\\ & & \cdot \left(\frac{\partial^{s_{1}+\cdots +s_{m}}A_{i}^{l}}{\partial \Theta_{1}^{s_{1}}\cdots \partial \Theta_{m}^{s_{m}}}\right)\left(\Theta_{1}^{k},\cdots ,\Theta_{m}^{k}\right) \end{array}$$ (12)

and then address the linear part of the approximation by

$$\begin{array}{ccc} A_{i}^{l}\left(\Theta^{l}\right)-A_{i}^{k}\left(\Theta^{k}\right) & = & \sum_{\alpha }\left(\Theta_{\alpha }^{l}-\Theta_{\alpha }^{k}\right)\frac{\partial A_{i}^{k}\left(\Theta \right)}{\partial \Theta_{\alpha }^{k}}\\ & = & \sum_{\alpha }\left(\Theta_{\alpha }^{l}-\Theta_{\alpha }^{k}\right)p_{i\alpha }^{k} \end{array}$$ (13)

in order to estimate $p_{i\alpha }^{k}$ from the linear regression using Eq (13).

After fixing the value of $p_{i\alpha }^{k}$, $\beta_{ij\alpha }^{k}$ is calculated using the same strategy. Because $p_{i\alpha }^{k}$ is the multivariate function of $A_{j}^{k},j=1,2,\cdots ,n$ and the environmental parameters Θ_*α*_, the difference between $p_{i}^{k}\left(\Theta^{k}\right)$ and $p_{i}^{l}\left(\Theta^{l}\right)$ can be also expressed using the full version of Taylor expansion.

$$\begin{array}{ccc} p_{i\alpha }^{l} & & =\sum_{r_{1}=0}^{\infty }\cdots \sum_{r_{d}=0}^{\infty }\sum_{s_{1}=0}^{\infty }\cdots \sum_{s_{m}=0}^{\infty }\frac{{\left(A_{1}^{l}-A_{1}^{k}\right)}^{r_{1}}\cdots {\left(A_{d}^{l}-A_{d}^{k}\right)}^{r_{d}}{\left(\Theta_{1}^{l}-\Theta_{1}^{k}\right)}^{s_{1}}\cdots {\left(\Theta_{m}^{l}-\Theta_{m}^{k}\right)}^{s_{m}}}{r_{1}!\cdots r_{d}!s_{1}!\cdots s_{m}!}\\ & & \cdot \left(\frac{\partial^{r_{1}+\cdots +r_{d}+s_{1}+\cdots +s_{m}}p_{i\alpha }^{l}}{\partial A_{1}^{r_{1}}\cdots \partial A_{d}^{r_{d}}\partial \Theta_{1}^{s_{1}}\cdots \partial \Theta_{m}^{s_{m}}}\right)\left(A_{1}^{k},\cdots ,A_{d}^{k},\Theta_{1}^{k},\cdots ,\Theta_{m}^{k}\right) \end{array}$$ (14)

Hence, analogous to Eq (11), the linear part to describe $\beta_{ij\alpha }^{k}$ based on two samples *k* and *l* is given by:

$$\begin{array}{ccc} p_{i\alpha }^{l}-p_{i\alpha }^{k} & = & \sum_{j}\left(A_{j}^{l}-A_{j}^{k}\right)\frac{\partial p_{i\alpha }^{k}}{\partial A_{j}^{k}}+\sum_{\alpha }\left(\Theta_{\alpha }^{l}-\Theta_{\alpha }^{k}\right)\frac{\partial p_{i\alpha }^{k}}{\partial \Theta_{\alpha }^{k}} \end{array}$$ (15)

here, the first part in Eq (14) addresses the change of other species abundances, whereas the second part addresses the change of environmental parameters. Analogous to Eq (13), the linear regression can be used to estimate $\frac{\partial p_{i\alpha }^{k}}{\partial A_{j}^{k}}$. Based on the Eq (9), the values of $\beta_{ij\alpha }^{k}$ are calculated as

$$\begin{array}{c} \beta_{ij\alpha }^{k}=\frac{\partial p_{i\alpha }^{k}}{\partial A_{j}^{k}}\frac{1}{A_{i}^{k}} \end{array}$$ (16)

The terms $\frac{\partial p_{i\alpha }^{k}}{\partial \Theta_{\alpha }^{k}}$ can be estimated also from Eq (15). These by-products address the influence from the environmental parameter change on the change of $p_{i\alpha }^{k}$. Although they do not provide information on the interaction between species *i* and *j*, they may provide valuable information of the interaction value $\beta_{ij\alpha }^{k}$.

In case that $\frac{\partial p_{i\alpha }^{k}}{\partial \Theta_{\alpha }^{k}}$ is of no interest, the model Eq (14) can be reduced to a simplified version by making use of only the first part in each Eqs (14) and (15) to then calculate $\beta_{ij\alpha }^{k}$ using Eq (16). This simplified version represents a different model of interaction calculation.

All the numerical calculations above are based on the linear part of the Taylor expansion. In order to cope with potential nonlinear properties of the data, it is also possible to include the higher order terms in the linear regression model. For example, when the second order terms are added into the Eq (13)

$$\begin{array}{ccc} A_{i}^{l}\left(\Theta^{l}\right)-A_{i}^{k}\left(\Theta^{k}\right) & = & \sum_{\alpha }\left(\Theta_{\alpha }^{l}-\Theta_{\alpha }^{k}\right)\frac{\partial A_{i}^{k}\left(\Theta \right)}{\partial \Theta_{\alpha }^{k}}+\frac{1}{2}\sum_{\alpha \beta }\left(\Theta_{\alpha }^{l}-\Theta_{\alpha }^{k}\right)\left(\Theta_{\beta }^{l}-\Theta_{\beta }^{k}\right)\frac{\partial^{2}A_{i}^{k}\left(\Theta \right)}{\partial \Theta_{\alpha }^{k}\partial \Theta_{\beta }^{k}} \end{array}$$ (17)

the resulting Eq (17) will allow performing numerical calculations by additionally including the nonlinear part. However, the numerical calculations will substantially increase in complexity.

The models introduced allow different levels of precisions (Eqs (10)–(17)). The user can choose any of these based on the defined preferences, e.g., numerical precision of the input data or also the availability of computational power.

### Data structure and precision of calculation

Data sparsity is the first issue which needs to be taken into account. In Eq (16), term $A_{i}^{k}$ is in the denominator. If the species has not been observed and hence has the abundance value 0 in one sample, this species cannot be included in the mathematical treatment. Therefore, abundance values of 0 have to be removed before interaction analysis.

The second important issue is the data type of abundance *A*_*i*_. For several organismic groups such as most plants and animals absolute abundance values *A*_*i*_ can be determined for each taxon. In contrast, a taxon-wise determination of absolute abundances is technically hardly feasible for bacterial microbes. Microbial communities are typically assessed by metagenomic high-throughput sequence data which yield relative abundances of taxa. However, since the overall cell numbers of microorganism in many cases do not change to a large extent, changes in absolute abundances are expected to be less pronounced than those in relative composition.

The structure of relative abundance data is characterized by an intrinsic compositional effect. This could produce misleading results in correlation analysis and would not reflect the true correlation as would have been the case for absolute abundance values [26–31]. As the sum of relative species abundance values by definition is constrained to 1, these values are not independent of each other. Fluctuations in the relative abundance of one species have an effect on the relative abundance of the rest of the community without that the rest of the community may actually have changed in absolute abundance. For example, if the abundance of a dominant species (e.g. 95% of all species) changes, relative abundances of all other species vary in the opposite direction. This creates artificial negative correlations with the dominant species which would not be the case with absolute abundances. Compositional effects may be severe in some data sets but mild in others. For example, compositional effects are most pronounced in communities with low species richness and/or pronounced dominance structure. The *α* diversity (of the samples in question is, therefore, a good predictor of the strength of compositional effects [28]. To decrease the influence of compositional effects, a series of methods based on the log-transformed techniques was developed [26–29].

Our interaction approach is not only conceptually but also mathematically fundamentally different from a correlation analysis. For example, whereas correlation aims to maximize the recovery of covariance *cov*(*A*_*i*_, *A*_*j*_), our interaction approach aims to derive the correct partial derivative of abundance *A*_*j*_ with respect to the environmental parameters Θ and other species *A*_*j*_. Thus, compositional effects have to be treated differently, as we show in detail below.

First, we discuss the relationship between the absolute abundance and the relative abundance, and the difference in results when we did the interaction analysis on both types of abundance data.

In the formalism of the interaction analysis, the terms $\frac{\partial A_{i}^{k}\left(\Theta \right)}{\partial \Theta_{\alpha }^{k}}$, $\frac{\partial A_{i}^{k}\left(\Theta \right)}{\partial A_{j}^{k}}$ play the very important parts in the whole calculation. For generality, we suppose *y*_*i*_ as the absolute abundance of species *i*, *x*_*i*_ as the relative abundance. We need to deduce the relationship between $\frac{\partial y_{i}}{\partial \Theta_{\alpha }}$ $\frac{\partial y_{i}}{\partial y_{j}}$ and $\frac{\partial x_{i}}{\partial \Theta_{\alpha }}$ $\frac{\partial x_{i}}{\partial x_{j}}$.

The relationship between *x*_*i*_ and *y*_*i*_ is

$$\begin{array}{c} x_{i}=\frac{y_{i}}{\sum_{\alpha }y_{\alpha }} \end{array}$$ (18)

calculate the derivative on both sides of Eq (18), we have

$$\begin{array}{c} \partial x_{i}=\frac{\partial y_{i}\sum_{\alpha }y_{\alpha }-y_{i}\sum_{\alpha }\partial y_{\alpha }}{{\left(\sum_{\alpha }y_{\alpha }\right)}^{2}} \end{array}$$ (19)

Therefore, we have

$$\begin{array}{ccc} \frac{\partial x_{i}}{\partial \Theta } & = & \frac{\frac{\partial y_{i}}{\partial \Theta }\sum_{\alpha }y_{\alpha }-y_{i}\sum_{\alpha }\frac{\partial y_{\alpha }}{\partial \Theta }}{{\left(\sum_{\alpha }y_{\alpha }\right)}^{2}}\\ & = & \frac{\frac{\partial y_{i}}{\partial \Theta }}{\sum_{\alpha }y_{\alpha }}-\frac{y_{i}\sum_{\alpha }\frac{\partial y_{\alpha }}{\partial \Theta }}{{\left(\sum_{\alpha }y_{\alpha }\right)}^{2}} \end{array}$$ (20)

$$\begin{array}{ccc} \frac{\partial x_{i}}{\partial x_{j}} & = & \frac{\partial y_{i}\sum_{\alpha }y_{\alpha }-y_{i}\sum_{\alpha }\partial y_{\alpha }}{\partial y_{j}\sum_{\alpha }y_{\alpha }-y_{j}\sum_{\alpha }\partial y_{\alpha }}\\ & = & \frac{\left(\sum_{\alpha }y_{\alpha }-y_{i}\right)\frac{\partial y_{i}}{\partial y_{j}}-y_{j}\sum_{\alpha \neq i}\frac{\partial y_{\alpha }}{\partial y_{j}}}{\sum_{\alpha }y_{\alpha }-y_{j}\frac{\partial y_{i}}{\partial y_{j}}-y_{j}\sum_{\alpha \neq i}\frac{\partial y_{\alpha }}{\partial y_{j}}} \end{array}$$ (21)

When ∑_*α*_ *y*_*α*_ > > *y*_*α*_, diversity of the species is high. The term $\frac{y_{i}\sum_{\alpha }\frac{\partial y_{\alpha }}{\partial \Theta }}{\sum_{\alpha }y_{\alpha }}$ approaches zero. Eq (19) reduce to $\frac{\partial x_{i}}{\partial \Theta }\approx \frac{\frac{\partial y_{i}}{\partial \Theta }}{\sum_{\alpha }y_{\alpha }}$. This approximation reveals that the effect of the environmental parameter Θ on the rate of change of relative abundance is different from the corresponding rate of change of absolute abundance by a factor $\frac{1}{\sum_{\alpha }y_{\alpha }}$. This factor has a positive value, and will keep the sign of $\frac{\partial x_{i}}{\partial x_{j}}$ $\frac{\partial y_{i}}{\partial y_{j}}$ the same. Similarly, Eq (21) reduce to $\frac{\partial x_{i}}{\partial x_{j}}\approx \frac{\partial y_{i}}{\partial y_{j}}$. This approximation means that the effect of the species *j* on the rate of change of relative abundance of species *i* is roughly the same as the corresponding rate of change of species absolute abundance of the species. Therefore, the interaction analysis based on the relative abundance can be roughly approximate to the corresponding analysis based on the absolute abundance. However, in the case of a lower species diversity, the upper approximation will not exist anymore, and the relation between the relative abundance and absolute abundance will become complex. Since in most cases microbial communities are highly diverse, our interaction analysis is expected to yield reasonable results. Even for lower diversity, interaction analysis of relative abundance data can yield useful data for understanding ecosystem properties.

Actually, the compositional effect has its basis in the non-independence of the relative abundance. In order to decrease the effect of non-independence, a robust algorithm is needed for the numerical calculations. The precision and robustness of our numerical calculation depend on the linear regression estimates (Eqs (11)–(13), etc.). The least squares estimation (function stats::*lm*() in the R language) is not a robust algorithm since it is very sensitive to the initial input data. Therefore, we applied the more robust maximal likelihood estimation instead (R function MASS::*rlm*()). However, this algorithm has some requirements: input data should not have singularity, i.e. no linear relationship (collinearity) among the columns of the input data matrix [32, 33]. Therefore, the test for singularity on both relative abundance data and environmental parameters data needs to be performed before regression analysis. If the input data have collinearity, the algorithm will remove one species or one environmental parameter randomly, and repeat the test for singularity in the new data sets until all the collinearity relationships are removed. This pretreatment not only improves the robust numerical calculation but also decreases the compositional effects.

Another issue related to the precision of calculation is the relationship between the samples number and the number of variables. Sample number should be larger than the number of variables to avoid indeterminate equations or overfitting. Other suggestions to avoid overfitting which are not used in our methods are discussed in [34–36].

### Summarizing $\beta_{ij\alpha }^{k}$ into the global interaction level *β*_*ij*_

The interaction level $\beta_{ij\alpha }^{k}$ has four indexes *i*, *j*, *α*, *k*, which refer to a specific pair of taxa *i*, *j*, a specific environmental parameter *α* and a specific sample *k*. For each pair of species *j* with interaction influence on species *i*, there is a two-dimensional matrix of numerical $\beta_{ij\alpha }^{k}$ values with *α* rows and *k* columns. In either row or column, the values can be either positive, negative or zero. Positive values indicate a positive influence, negative values indicate a negative influence. Values may be non-normal distributed including extreme outlier values. To summarize these results into a more global interaction level value *β*_*ij*_ between species *i* and species *j*, we suggest the following different methods which can be chosen based on user preference.

Prior to any summarizing approach, users may decide to give different weight to $\beta_{ij\alpha }^{k}$ values for different environmental parameters, based on some prior knowledge about Θ_*α*_. We estimate $\beta_{ij}^{k}$ by performing a linear combination of $\beta_{ij\alpha }^{k}$ across all the environmental parameters:

$$\begin{array}{c} \beta_{ij}^{k}=\sum_{\alpha }\left(C_{\alpha }\times \beta_{ij\alpha }^{k}\right) \end{array}$$ (22)

where *C*_*α*_ is the applied weight of a given environmental parameter *α*. Prior knowledge on *C*_*α*_ can be obtained from, e.g. multivariate statistics. A Redundancy Analysis (RDA) allows determining those environmental parameters which significantly contribute to an observed community composition. The eigenvalue for each Θ_*α*_ in RDA analysis can be used as *C*_*α*_ weight. The derived weighted $\beta_{ij\alpha }^{k}$ values can be summarized in the same way as the original $\beta_{ij\alpha }^{k}$ values using the methods suggested below.

The most straightforward way is to summarize $\beta_{ij\alpha }^{k}$ estimates by standard summarizing statistics (mean, median, maximum, minimum). This approach retains the strength and direction of the interaction.

It is not recommendable, to sum up to $\beta_{ij\alpha }^{k}$ values, as positive and negative values could equal out each other resulting in a rather low *β*_*ij*_ value. In case the user is interested in a sum value, the standard norm definition could be applied. Note that this is possible only at the expense of losing information about the direction of interaction, as only positive values will be obtained. For example,

$$\begin{array}{c} \beta_{ij}^{k}=∥\left(\beta_{ij\alpha }^{k}\right)∥=\sqrt{\sum_{\alpha }{\left(\beta_{ij\alpha }^{k}\right)}^{2}} \end{array}$$ (23)

represents a summed $\beta_{ij\alpha }^{k}$ interaction level between species *i* and species *j* across all environmental parameters *α* at sample *k*. Using the same formula as in Eq (23), several other quantities could be determined, e.g., *β*_*ij*_ would represent a summed $\beta_{ij}^{k}$ across all samples *k*. Similarly, $\beta_{i}^{k}$ represents the summed $\beta_{ij}^{k}$ across all cases where *j* ≠ *i*. Finally, *β*_*i*_ represents the summed $\beta_{i}^{k}$, which is the global influence of interaction from all the other species on species *i* across all the samples *k*.

Neither of the summarizing statistics addressed above captures the center of $\beta_{ij\alpha }^{k}$ values for a given environmental parameter *α* across all samples *k* appropriately and might therefore not yield the necessary insight into the interaction structure. A curve fitting approach including bootstrapping on $\beta_{ij\alpha }^{k}$ values with subsequent peak value extraction, as implemented in the eHOF R package [37] would be appropriate but could be computationally demanding with increasing taxa and environmental parameter numbers. As a compromise, we extract the median of those values which are represented in the peak from a $\beta_{ij\alpha }^{k}$ density distribution. The peak values, one per environmental parameter *α* for each species pair *ij* or *ji* and denoted therefore as *M*_*α*_, could be summarized using the above standard summarizing statistics but would also suffer from the same shortcomings.

We, therefore, propose a custom approach which focuses on the dominant patterns of direction and strength of $\beta_{ij\alpha }^{k}$ values. The result will be a conservative estimate of direction and strength of *β*_*ij*_ values reflecting the dominant interactions between species *i* and species *j*. This procedure is based on two steps and may involve several user-based definitions of applied threshold values.

Firstly, the direction of interaction by categorizing $\beta_{ij\alpha }^{k}$ values is determined for each *α* across all samples *k* as being either positive or negative. In case the majority (we use 80%) of all $\beta_{ij\alpha }^{k}$ of a given *α* belongs to either category, the direction of interaction is classified either as positive or negative, respectively. In case that no preponderance can be identified, the given *α* does not contribute to a global *β*_*ij*_ determination and hence is ignored in the further analysis. The above peak determination approach yields a set of *M*_*α*_ values along with robust assignment of direction (either positive or negative).

Secondly, the set of *M*_*α*_ values per each taxon pair *ij* or *ji* is used to yield a global interaction value *β*_*ij*_ or *β*_*ji*_, respectively. Note that *M*_*α*_ values are characterized by a direction (positive or negative) and by a certain strength (magnitude of the numerical value). Depending on the type of distribution of both direction and strength of value, two different ways for further evaluation can be taken into account. In case that the majority (we use 80%) of *M*_*α*_ values can be assigned to either direction, the respective *M*_*α*_ values are summarized by determining the median value, which represents then the global *β*_*ij*_ across all *α* parameter and *k* samples and is additionally characterized by a specific direction (positive or negative). Note that based on users interest, any other majority threshold value and summarizing statistic such as mean, minimum, or maximum can also be taken into account. However, in case that no preponderance can be identified, a decision based on the above majority rule on direction can not be taken and will be replaced by a decision based on the magnitude of *M*_*α*_ values. We determine for each group of positive or negative *M*_*α*_ values the respective median values *M*_*α*+_ and *M*_*α*−_ values. In case the ratio of absolute values of *M*_*α*+_ or *M*_*α*−_ value is larger than two, the direction of the interaction is assumed to be represented by the larger *M* value (either *M*_*α*+_ or *M*_*α*−_), with the respective *M* value being the global *β*_*ij*_ across all *α* parameter and *k* samples. If *M*_*α*+_ and *M*_*α*−_ have comparable absolute values and also have equal proportions of either positive or negative direction, it is concluded that it is not possible to determine a global *β*_*ij*_ across all *α* parameter and *k* samples for the specific species pair of *i* and *j*.

In sum, we have suggested several workflows summarizing $\beta_{ij\alpha }^{k}$ values into a global interaction value *β*_*ij*_ that also contains the information on the direction (positive or negative interaction). Note that the choice of methods and choice of settings of several threshold values for a decision on intermediate steps is dependent on user preferences.

### Robustness estimation of *β*_*ij*_ values

The magnitude and sign of the *β*_*ij*_ value depend on variables of the experimental data, such as variability of sampling site choice and the reliability (degree of precision) with which numerical values such as relative abundances of taxa or environmental parameter were determined [38]. We, therefore, implemented several methods to explore the robustness of the *β*_*ij*_ value (strength and direction) with respect to sampling site choice and numerical precision uncertainties in determining relative abundances and values of environmental parameter. Firstly, the effect of samples, which include values for both the environmental parameter and the relative abundances of taxa, is accessed by random sampling on soil samples. Secondly, we add numerical noise to either the original data of relative abundances or the environmental parameter values by randomly adding or subtracting error terms using formula

$$\begin{array}{c} \tilde{\Theta_{\alpha }^{k}}=\Theta_{\alpha }^{k}\left(1+\epsilon \right) \end{array}$$ (24)

where $\Theta_{\alpha }^{k}$ is the original environmental parameters matrix, $\tilde{\Theta_{\alpha }^{k}}$ is the perturbed data matrix, and *ϵ* is the error term. Values for *ϵ* are generated as follows:

$$\begin{array}{c} \epsilon =U(-1,1)e \end{array}$$ (25)

where *U*(−1, 1) is the uniform distribution in the range [−1, 1] and *e* is the error level. The height of the error level, given as the proportion of the original value, e.g. 0.01% to 50%, can be defined by the user.

Similarly, random perturbations can be added to the numerical values of the species abundances. Values of *β*_*ij*_ obtained in repeated runs of data perturbation are then summarized using monovariate statistics (mean, 95% confidence interval, null hypothesis testing).

## Application and results

We tested our method on datasets obtained from grassland soils of the German Biodiversity Exploratories (http://www.biodiversity-exploratories.de; [39]). The sampling plots were located in three regions in Germany: Schorfheide-Chorin (Schorfheide Exploratory; SE) in Brandenburg, national park Hainich-Dün (HE) in Thuringia, and biosphere reserve Schwäbische Alb (AE) in Baden-Württemberg. In every region, 50 grassland sites with different land-use intensities were investigated in the year 2011, resulting in a total of 150 plots analyzed in this study. At each plot aboveground plant parts in grasslands were removed before fourteen soil cores (diameter, 5 *cm*) were taken from the upper 15 *cm* of the A horizon from a 20 × 20 *m* subarea. The 14 samples were combined, homogenized and 10*g* of the homogenized soil were frozen immediately in liquid nitrogen and stored until nucleic acid extraction for the determination of relative abundances of prokaryotic (RNA extraction) and fungal and protist (DNA extraction) communities by high-throughput sequencing.

The original test data encompasses 14 environmental parameters which were determined for each sieved (2 *mm*) soil sample. These are pH, soil moisture (%), nitrate (${\text{NO}}_{3}^{-}$), ammonium (${\text{NH}}_{4}^{+}$), mineral nitrogen (N), microbial C [all values as *μ* ⋅ *g* soil^−1^], organic and inorganic carbon (C) [all values as *mg* ⋅ *g* soil^−1^], fine root biomass [*g* ⋅ *cm*^−3^], total N and C in roots (%), C/N ratio in soil, microbial C/N ratio, and C/N ratio in roots. The test for singularity (QR decomposition [40]) finds a rank of the environmental parameters matrix of 13 due to the collinearity between ${\text{NO}}_{3}^{-}$ and ${\text{NH}}_{4}^{+}$. This means that either ${\text{NO}}_{3}^{-}$ or ${\text{NH}}_{4}^{+}$ should be removed. Therefore, the interaction analysis is done with only a set of 13 environmental parameters after removing ${\text{NH}}_{4}^{+}$. Details on nucleic acid extractions, high-throughput sequencing, taxonomic classification, and determination of physicochemical soil parameters have been published elsewhere [41–45].

Calculations were performed for 17 taxonomic groups at the level of phylum or classes which represent abundant groups. The estimation of their relative abundances is generally of high precision (typically less than 5% deviation [23]). $\beta_{ij\alpha }^{k}$ and $\beta_{ji\alpha }^{k}$ of each pair of species *i* and *j* were determined for each soil sample *k* and for each environmental parameter *α*.

Overall, 150 samples, 17 taxonomic groups, and 13 environmental parameters were included in the data analysis. This data structure satisfies the requirement of sample size which is discussed in section. We scaled the species abundance and environmental parameters data (variance equals 1, uncentralized) to avoid the problem of large difference scale in different variables.

### The degree of interaction changes with the gradient of the environmental conditions

**Fig 2:**
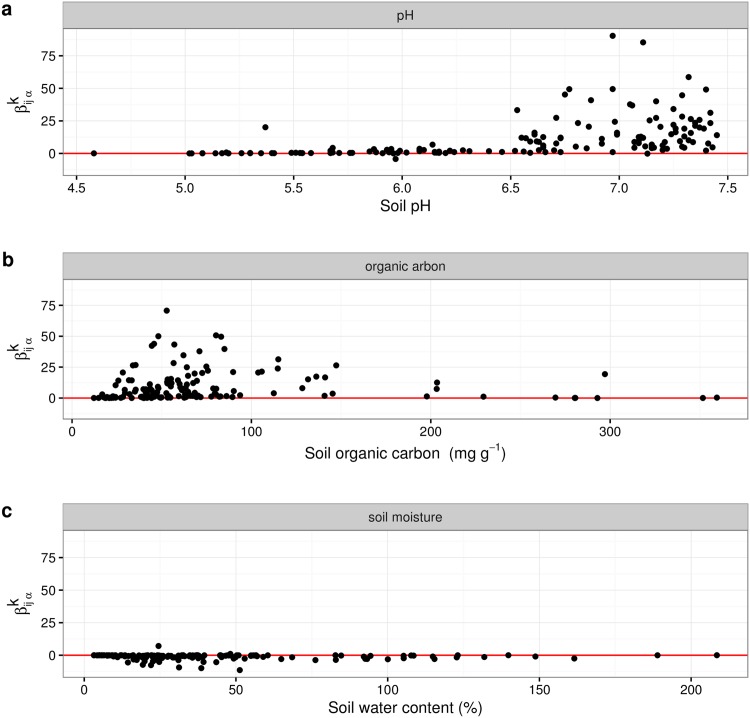
The distribution of $\beta_{ij\alpha }^{k}$ along the environmental gradient.

The Fig 2 exemplifies the change of $\beta_{ij\alpha }^{k}$ estimates across the environmental gradient of three soil parameters for the interaction influence of acidobacterial subgroup Gp3 on acidobacterial subgroup Gp1. Y-axis range is the same for all three subfigures.

Whereas for pH and organic carbon a strong positive interaction is predicted, soil moisture suggests a weak negative impact. Notably, the strength of interaction varies along the gradient of the environmental parameter and increases substantially above a pH value of 6.5. In contrast, the interaction strength along gradients of organic carbon and soil moisture appears to be larger at rather low values of organic carbon and soil moisture respectively. In sum, $\beta_{ij\alpha }^{k}$ values may vary substantially across the gradient of environmental parameters.

### Comparison between the global interaction matrix and the correlation matrix

**Fig 3:**
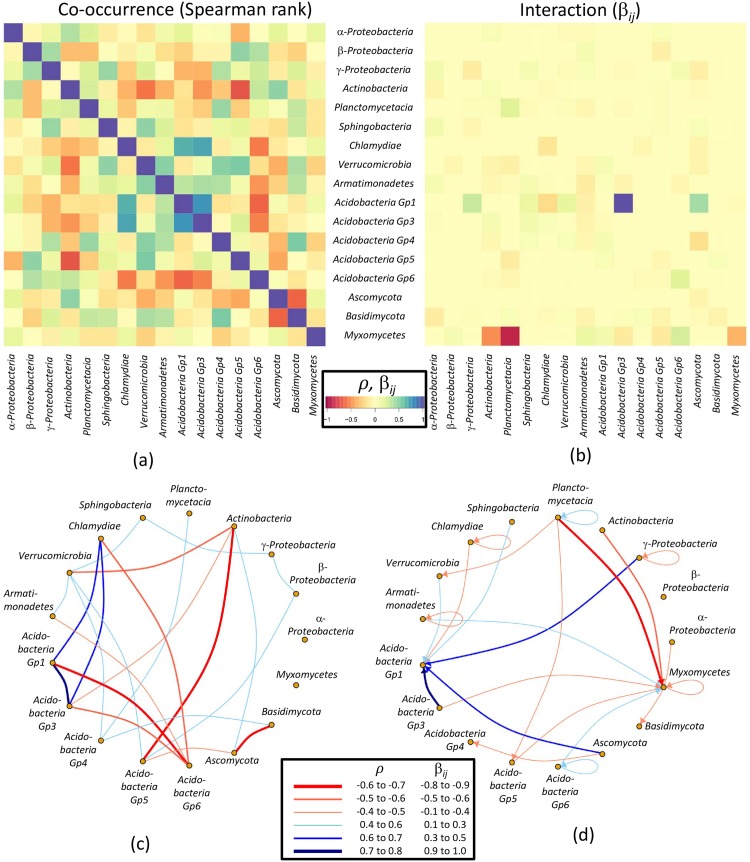
The comparison between the correlation and interaction analysis.

The $\beta_{ij\alpha }^{k}$ and $\beta_{ji\alpha }^{k}$ values were then summarized by global *β*_*ij*_ and *β*_*ji*_ values, respectively, to estimate (a) the direction of interaction, which can be either positive or negative, and (b) the strength of interaction. In addition, the global interaction values were compared to results of standard co-occurrence analyses calculated by means of a Spearman rank correlation matrix *C*_*ij*_ based on relative abundances of the taxa. Both sets of results were also visualized as networks which displayed the dominant patterns (for values -0.1 > *β*_*ij*_ > 0.1; -0.4 > *ρ*_*Spearman*_ > 0.4) (Fig 3).

Fig 3 provides the heatmaps (a,b) and network figures (c,d) of Spearman rank correlation matrix (a,c) and interaction matrix (b,d). Taxa depicted on the x-axis (columns) have interaction influence on taxa on the y-axis (rows). The red and blue colors indicate the negative and positive effects, respectively. For example, acidobacterial subgroup Gp3 has a high positive interaction influence on acidobacterial subgroup Gp1, whereas the bacterial *Planctomycetacia* have a strong negative interaction influence on protist group of *Myxomycetes*. Note that the heatmap color code is the same for both *β*_*ij*_ and Spearman *ρ* values. In the network figures, low Spearman *ρ* and low *β*_*ij*_ are not shown (but displayed in the heatmap), whereas the remaining values were artificially grouped into different categories (see color legend networks). Note that the interaction network displays also the direction of interaction by arrows, whereas correlation analysis does not enable any statement of directionality.

Important characteristics of interaction estimates and their difference to co-occurrence estimates are highlighted in Fig 3.

Firstly, taxa involved in multiple co-occurrences are not necessarily involved in corresponding interactions and vice versa. For example, acidobacterial subgroups Gp3, Gp5, Gp3 and also *Actinobacteria* share numerous co-occurrences with other taxa but are far less involved in interactions. Similarly, *Myxomycetes* and acidobacterial subgroup Gp1 both show numerous interactions to other taxa, but are less, if at all, involved in correlations.

Secondly, in case that two taxa are characterized by both strong correlation and interaction, it is not possible to predict from the type of correlation on the direction of interaction and vice versa. For example, both acidobacterial subgroup Gp3 and the *Chlamydiae* show strong positive correlations with each other and with acidobacterial subgroup Gp1. A strong positive interaction is observed only from Gp3 to Gp1, whereas the interactions of *Chlamydiae* on Gp1 are weakly negative and on Gp3 only very weak (*β*_*ij*_ = 0.04).

Thirdly, whereas co-occurrences within the same taxon are always positive at *ρ* = 1 (see heatmap, but not depicted in the network), overall interactions of taxa with itself can be both negative and positive. For example, *Myxomycetes* and *Chlamydiae* appear to have a negative interaction on themselves, whereas acidobacterial subgroup Gp6 and *Plancomycetacia* appear to have a positive influence on its own. Mathematically, this can be explained by analogy to the species self-effect in logistic equations, in which the rate of change of species abundance has also an influence in itself. In other words, this interaction value can be treated as the leading order of the solitary part in Eq (1). When the rate of change *p*_*i*_ decreases with the increase of its abundance *A*_*i*_, the interaction influence from itself will be negative. In the converse situation, the influence will be positive. As a biological interpretation, taxa negatively interacting with each other (as implied here by negative *β*_*ij*_ values) have reached the carrying capacity within their ecological niche. Alternatively, these results could be a consequence of hierarchically nested taxa that are strongly interacting with each other, resulting in a cumulative positive or negative interaction of the higher level taxon on its self (e.g. *Chlamydia*).

Finally, it appears as if taxa are preferentially either exerting or experiencing interaction influence. For example, *Myxomycetes* share a lot of mostly negative interactions with other taxa, however, in all cases *Myxomycetes* are being influenced by others but are not exerting influence on others. Antibiotics production of bacteria could be a likely explanation [46]. The same is true for acidobacterial subgroup Gp1, which is, mostly positively, under interaction influence by other taxa. Only few taxa appear to both, experience as well as exert effects through influence (*Verrumicrobia*, *Acidobacteria* subgroup Gp5).

### Estimation of robustness on the interaction influence calculation

In order to estimate the robustness of *β*_*ij*_ with respect to the numerical imprecision of the input data, we performed several perturbation assays. For this, we chose six examples of global *β*_*ij*_ interaction values from the Fig 3 which are representative of different strengths of interaction values with both a positive or a negative direction. Following the distribution of *β*_*ij*_ shown in the interaction heatmap of Fig 3, we tested larger, median, and low *β*_*ij*_ values for their robustness on data perturbations. The effect of variation in sample composition on *β*_*ij*_ is evaluated by 1000 iterations of randomly sampling 90% of the samples without replacement. We refrain from using the classical bootstrapping (sampling with replacement), as the deviation term (Eq (11)) will turn zero for twice or more of subsampled data and hence will be of no informative value in the downstream regression analysis (Eq (11)).

The effect of either numerical precision of environmental parameter values or relative abundances of taxa was evaluated by randomly adding or subtracting error terms (0.01%, 0.1%, 5%, 10%, 20% and 50%) to the original values. The effect of both numerical precision of environmental parameter values and relative abundances of taxa was evaluated by randomly adding or subtracting error terms (5%, 20%) to the original values.

**Fig 4:**
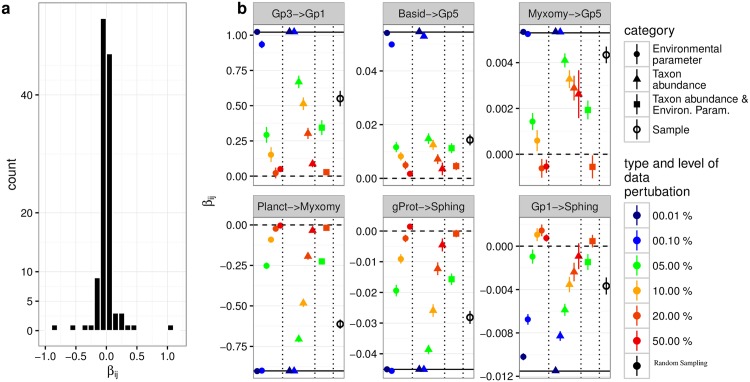
The test of robustness.

Each 1000 iterations were performed for each error term and data type. We analyzed the data by means of comparison of 95% confidence interval, which provide information on effect sizes additionally to null hypothesis significance testing [47]. The robustness of *β*_*ij*_ estimations at different levels of data perturbation. are presented in Fig 4.

The plot (a) presents the distribution of *β*_*ij*_ as shown in the interaction heatmap in Fig 3. The plot (b) shows the robustness estimations on exemplary positive (upper row) and negative (lower row) *β*_*ij*_ values of decreasing strength (from left to right) taken from the interaction heatmap in Fig 3. The respective interactions from taxon *j* on *i* are listed as abbreviations in the panel header (Gp1 and Gp3: acidobacterial subgroups Gp1 and Gp3; Basid: *Basidiomycetes*; Myxomy: *Myxomycetes*; Planct: *Planctomycetacia*; gProt: *γ*-*Proteobacteria*; Sphing: *Sphingobacteria*). Only the strong interactions (left panels in (b) are depicted in the interaction network in Fig 3). The black horizontal line indicates the original *β*_*ij*_ values. Dots and vertical lines represent mean and 95% confidence interval bars from 1000 iterations of each type of data perturbation (see color legend). Horizontal dashed lines separate perturbations on environmental parameter values, relative taxon abundances, and sampling sites. Very small 95% CI values are are not visible as they are covered by the size of the point estimate dot (mean value of 1000 iterations).

Note, however, that in all cases where the 95% CI bar did not cross the zero line, p was < 0.01 in a two-sided one-sample t-test.

Typically, the 95% CIs are very small, suggesting the algorithm for numerical calculations to be robust. However, with increasing error level (from 0.01% to 10%) the 95% CIs become larger. This can be explained by the accumulated error in the numerical calculation and the nonlinear structure of the data. In our model, we use the linear part of the Taylor expansion as the approximation, and the numerical calculation is based on the linear regression. At small error level, the Taylor expansion can be reliably estimated by its linear part. At increasing error level, the potentially nonlinear structure of the data will become more relevant and therefore may generate increasing uncertainty in the estimation. Principally, this issue could be solved by extending the Taylor expansion to higher orders to take into account the nonlinear structure of the data.

The majority of *β*_*ij*_ values was very small for both positive and negative directions (plot a in Fig 4). This is the result of our conservative custom approach for *β*_*ij*_ summarizing, which is based on the peak values of $\beta_{ij\alpha }^{k}$ density distributions and which is typically close to zero. However, Fig 2 indicates that individual $\beta_{ij\alpha }^{k}$ values can be considerably larger than 1.

There is a substantial effect of increasing error term size on the reduction of the original *β*_*ij*_, which appears to be much larger than the effect on the increase of 95% CI intervals with increasing error term (figure b in Fig 3). This finding is independent of direction and strength of the original *β*_*ij*_ value and suggests that conclusions on direction and strength of interactions, especially in comparison of different pairs of taxa to each other, appear to be stable in the light of moderate error rates (up to 10%). The overall effect of error term size on data perturbations is larger for environmental parameter values than for values for the relative abundances of taxa. As a result, at larger error rates of environmental parameter values, the direction of interaction may change, suggesting that biological interpretation of very low *β*_*ij*_ should be treated with caution.

*β*_*ij*_ resulting from random sampling on soil samples are at comparable levels to *β*_*ij*_ resulting from 5% to 10% error term data perturbations on relative abundance values. Obviously, variation in the composition of samples does not change the estimates and the algorithm remains robust.

## Discussion

Our first application of the methods developed in the present study to real-world data allowed us to identify several biotic interactions that are likely to shape soil microbial communities but were previously not recognized. Notably, the novel approach can be used to resolve the full spectrum from intra-specific, over intra-phylum, to inter-domain interactions in complex microbial communities.

In our approach, environmental parameters which are not quantified in a study but which are nevertheless relevant for the interaction of species *i* on *j* will affect the results and hence the inference of biotic interactions. By comparison, lack of quantitative information on environmental parameters does not affect the results of co-occurrence patterns, where the correlation value of species *i* and *j* will stay the same irrespectively of any environmental parameter that has been determined or not. Yet, the relevance of unknown parameters that might control the correlation instead of direct biotic interactions will not be revealed by co-occurrence analysis. Increasing the number of organismic groups and environmental parameters in our type of analysis ultimately will require some data reduction approaches. The choice of which taxa and which environmental parameter should be included requires independent knowledge about their potential relevance. Such knowledge could be retrieved from, e.g., multivariate statistics.

The novel approach to estimate the strength and direction of biological interactions among taxa provides several advantages.

Firstly, our approach does not require repeated measurements at different time points. This is in contrast to approaches based on the discrete-time Lotka–Volterra model which requires concrete differential equation models [9, 48–50]. Often, these interactions are analysed within a predator-prey framework. However, the sampling of microbial communities is typically conducted in a destructive manner, which renders reproducible sampling of heterogeneous soil environment rather difficult or even impossible [43]. Instead, many soil microbial studies use cross-sectional format by taking multiple samples from different sites in parallel at the same time [51–53]. Our approach is far more general than the discrete-time Lotka–Volterra models employs derivations from the multivariate Taylor expansion function, and therefore allows to assess interactions using comparative cross-sectional datasets.

Secondly, our approach allows analysing interactions between two species *i* and *j* in the presence of other taxa *x*, *y*, *z* which may affect the interaction of species *i* on *j* [54, 55]. Analysing interactions between species i and j within a more complex community has already been addressed in discrete-time Lotka-Volterra models [9]. In our model, the user can deliberately remove species *x*, *y*, *z* to analyse the effect of their presence or absence on the interaction of species *i* on *j* or identify whether interactions appear stable despite varying community compositions. As an example, we observed that Acidobacteria subgroup Gp1 is apparently influenced by several other taxa (Fig 3). Using slightly different community compositions and slightly different sets of the environmental parameter, we observed that (i) Acidobacteria subgroup Gp1 remains being influenced by numerous other groups and that (ii) the observation of a strong positive interaction of Acidobacteria subgroup Gp3 on Gp1 remains (data not shown). The ecological function of Acidobacteria subgroup Gp1 is still largely unknown, but its involvement in several rather strong interactions suggests that they represent a keystone group of bacteria.

Thirdly, we can address interaction in the light of qualitatively and quantitatively different environmental parameters. Analogous to studying the effect of species composition, the user will be able to study the effect of a specific environmental parameter by removing it from the data set or by testing different combinations. More detailed analyses could be undertaken by determining *β*_*ij*_ separately for different ranges of environmental parameter values, e.g. at low versus high values of either pH, soil moisture, or land use intensities.

Fourthly, the results from our interaction calculations serve not only for biological interpretations but can be further used in statistical or modeling approaches. Quantities such as $\left(p_{i\alpha }^{k}\right)$, $\left(\beta_{ij\alpha }^{k}\right)$, $\left(\beta_{ij}^{k}\right)$, (*β*_*ij*_), $\left(\beta_{i}^{k}\right)$ and (*β*_*i*_) could be used in multivariate statistics or to construct dynamical equations within the framework of system stability analysis [56–58].

Finally, whereas in co-occurrence analysis it is not possible to distinguish between effects of true interaction and effects of similar response to environmental parameters without actually interacting, our approach enables to address separately the effects of biotic interactions and the abiotic response to the environmental parameter by using Eq (14).

At present, our model does not incorporate specific assumption about the mathematical expression of *S*_*i*_, *I*_*ij*_ in Eq (1). In the future work, the Monod equation or logistic functions could be included to update our model to a concrete form. The numerical calculation strategies to do the interaction estimation would not necessarily be affected by this.

Based on the quantification of the strength of interaction and the prediction of its direction that is provided by our new approach, the underlying mechanisms of interaction will have to be determined by complementary experimental approaches. For example, a strong negative interaction could be exerted via direct predation [55], antibiotics [59], or other types of chemical warfare such as volatiles [60]. Here, strong *β*_*ij*_ that link taxa which previously were not under suspect to interact under natural conditions could serve as models for future investigations of the interaction mechanisms. This will require the availability of cultured isolates, however.

## Supporting information

S1 File R code and data sets. “Abundscale.Rdata” is the original species relative abundance data. “Parascale.Rdata” is the original environmental parameters data. Due to the long computation time on the original data, two shorten data sets “Abundscale_short.RData” and “Parascale_short.RData” are also provided for the fast test. “test_git.R” is the code for the analysis workflow on both of original data and the shorten data. “InteractionAnalysis_git.R” contains all the developed functions which are used in “test_git.R”.
(7Z) Click here for additional data file.
